# The Role of Dark Septate Endophytic Fungal Isolates in the Accumulation of Cesium by Chinese Cabbage and Tomato Plants under Contaminated Environments

**DOI:** 10.1371/journal.pone.0109233

**Published:** 2014-10-08

**Authors:** Ousmane Diene, Nobuo Sakagami, Kazuhiko Narisawa

**Affiliations:** 1 Direction de la Protection des Végétaux, Ministère de l’Agriculture et de l’Equipement Rural, Thiaroye, Dakar, Sénégal; 2 College of Agriculture, Ibaraki University, Ami-machi, Ibaraki, Japan; NERC Centre for Ecology & Hydrology, United Kingdom

## Abstract

Following the 2011 Fukushima Daiichi Nuclear Power Plant accident, the preservation of the food chain from radionuclides contamination has become of crucial importance. The potential of Dark septate endophytic fungi in the management of Cs accumulation in plants under contaminated environments was investigated using Chinese cabbage and tomato plants. Four endophytic fungal isolates of different species, i.e. *Pseudosigmoidea ibarakiensis* I.4-2-1, *Veronaeopsis simplex* Y34, *Helminthosporium velutinum* 41-1, and as yet unidentified taxon 312-6 were tested *In Vitro* in two levels of Cs (5ppm and 10ppm). On the plant growth, the inoculation of the selected DSEs to both Chinese cabbage and tomato resulted in an increased biomass of up to 82% and 122%, respectively compared to control (non-inoculated) plants. With regards to the Cs accumulation, it varied with the host plant considered. In Chinese cabbage, DSEs inoculation caused higher Cs accumulation in above ground plant parts, whereas in tomato, Cs accumulation decreased significantly with three of the isolates tested, i.e., *V. simplex* Y34, *P. ibarakiensis* I.4-2-1, and the as yet unidentified taxon 312-6 suggesting low-risk transfer on the above ground plants parts as a result of high and negative plant reactions rather than high and positive reactions as it is the case with Chinese cabbage. These results suggested that DSEs can be recommended for use with Chinese cabbage to enhance phytoremediation of Cs in surrounding contaminated areas. With tomato, DSEs can be recommended for decreasing the accumulation of Cs in plants under contaminated environments.

## Introduction

The 2011 Fukushima Daiichi Nuclear Power Plant accident dispersed radioactive cesium (Cs) into the environment [1], [2], so that land forest and fresh water ecosystems surrounding the Fukushima area in Eastern and northwest Japan have been heavily contaminated [3], [4]. In natural land forest ecosystems, this radiocesium accumulates in the deposited litter but also in the upper soil profile, including the humus layer [5], [6]. Wild edible mushrooms and other organically grown crops that rely on organic matter for nutrients [7] therefore become a potential risk to human health through exposure via the primary food chain [8], particularly within the metal-contaminant hyper accumulators such as Chinese cabbage and tomato, both grown in these contaminated areas in Ibaraki and Tohoku, respectively. As such, the preservation of these high accumulator crops from radionuclide accumulation becomes therefore of crucial importance.

Symbiotic microorganisms, such as mycorrhiza and dark septate endophytic fungi (DSE) are known to associate with most forest trees and crops [9], [10]. The potential of these microorganisms in the accumulation of metal contaminants, especially heavy metals, and radionuclide Cs has attracted much attention. Arbuscular mycorrhizal fungi (AMF), for example, showed high cesium accumulation, reaching 41.7% to 71.7% in the above-ground portion of three grass species inoculated with *Glomus mosseae* and *Rhizophagus irregularis* (*G. intraradices*) [11]. More recently, AMF showed the ability to improve both the biomass and Cs uptake of plants such as sunflower and cucumber [12], [13]. Contrary to mycorrhizas, the potential of these DSEs in the management of plant growth and Cs accumulation in high accumulator crops under contaminated environments remains widely untapped. DSEs are not very common in soil but are often associated with roots of many plants, forming “symbiotic” relationships [14]. They have little or no host specificity [10], [15] and can even live in symbiosis with non-mycorrhizal plants such as Chinese cabbage [16]. There was some striking variation among plant species, but Solanaceous and Brassica plants are known as plants with high affinity for DSEs with regards to the greatest number of DSE fungi with isolates obtained from Solanaceous and Brassica plant root compared to other plants [16], [17].

Interactions between DSEs and host plants have improved plant fitness through mechanisms such as nitrogen transfer and uptake of phosphorus, and nutrient provision to host plants [16], [18]. We predicted that DSEs would play key regulation roles in extreme environments, especially in Cs-polluted conditions.

We studied the interaction between DSEs and host plants in Cs-polluted environments using Chinese cabbage and tomato. Both crops are hyper accumulator vegetables and are cultivated in heavily polluted areas in Ibaraki and Tohoku prefectures. The former is a leafy vegetable consumed entirely and the latter is a fruity vegetable cultivated for fruit consumption.

## Materials and Methods

### Fungal isolates and plants materials

Four DSE isolates selected from the culture collection of the Laboratory of Microbial Ecology, Ibaraki University, were tested in this study. All the isolates belong to different species and include: *Pseudosigmoidea ibarakiensis* isolate I.4-2-1, *Helminthosporium velutinum* isolate 41-1, *Veronaeopsis simplex* isolate Y34, and as yet unidentified taxon 312-6.

As host plants, Chinese cabbage c.v. Musou and tomato c.v. Hausu Momotaro (Takii Seed, Kyoto, Japan), two easily grown plant species of the Brassicae and Solanaceae families, respectively, were chosen mostly for their known high affinity for DSEs.

### Fungal culture and plant inoculation

The liquid culture method, as in hydroponic culture, with filter paper platform, was used to carry out the study. Each 120×30 mm test tube (Iwaki, Japan) contained approximately 20 ml oatmeal medium (Oatmeal, 10 g L^−1^) enriched with nutrients [MgSO_4_.7H_2_O (Wako Chemical Ind., Osaka, Japan), 1 g L^−1^; KH_2_PO_4_ (Wako Chemical Ind.), 1.5 g L^−1^; Nature aid, 5.5 g L^−1^], supplemented with cesium (Wako, Chemical Ind.) at selected concentrations of 5 and 10 ppm.

Fungal isolates were grown for two weeks on the top of a filter paper platform placed in each test tube and incubated at 23°C. When a sufficient fungal colony was obtained, surface-sterilized 2-day-old seedlings of Chinese cabbage or tomato were transplanted (two per test tube, seven test tubes per replicate) on to the growing colony and the whole set was incubated for three weeks at 23°C with an 18 h∶ 6 h (L:D) photoperiod at 600 lux. The procedure was similar for controls and the only difference is there was no fungal inoculation and plants were simply supported by a small piece of oatmeal agar. These seedlings were harvested and oven-dried at 40°C for 48 hours and their dried weights were measured and compared to the control (non-inoculated) plants.

### Anatomic observation

To determine the effect of Cs on roots of the host plants inoculated with fungal isolates, 3-week-old tomato and Chinese cabbage seedling roots were observed using an optical microscope after they were washed, cross sectioned, and stained in 50% acetic acid solution containing 0.005% cotton blue under a light microscope (BX51; Olympus, Tokyo, Japan).

### Seedling ignition and XRF analysis

Each dried seedling sample was ignited using an As One Programmable Furnace (MMF series; As One Corporation, Osaka, Japan) as follows: 105 °C for 10 h, 350 °C for 2 h, and 550°C for 5 h. The resulting ash was collected, weighed, and the content (%) of each sample was calculated by dividing the ignited seedling weight by the dried seedling weight.

To determine the elements composition of the ash, X-ray fluorescence (XRF) analysis was performed. Firstly, ashes of 7 samples were placed in one Eppendorf tube and mixed using a needle. These mixed ash samples were set in a holder (designed for small powder samples), and X-ray fluorescence spectroscopy analyses were conducted using a Shimadzu Energy Dispersive Fluorescence X-ray Spectrometer Rayny EDX-700HS (Shimadzu Corp., Kyoto, Japan) as follows: Vacuumed, Collimator: 1 mm; Applied voltage: 2 channels at 15 kV (0–4.4 KeV) and at 50 kV (4.4–40 KeV); Time: 400 sec for each channel. X-ray fluorescence analysis was performed 3 times for 1 pooled ash sample.

The amount of Cs contained in seedlings (Cs accumulation) was estimated by multiplying the Cs concentration (ppm) and the mean of ash content.

### Data analysis

The mean dry biomass of each treatment was calculated and analyzed with one-way ANOVA. Differences among treatment means were detected with Tukey's honestly significant difference test.

The mean Cs content of each treatment was estimated and analyzed with one-way ANOVA. Differences among treatment means were detected with Fisher (5%) test.

The quantitative capability of XRF analysis using EDX-700HS was also confirmed by 2 standard plant samples (peach leaf and tea leaf).

## Results

### Effects of Cs on the biomass of DSE-treated Chinese cabbage and tomato seedlings

**Figure 1:**
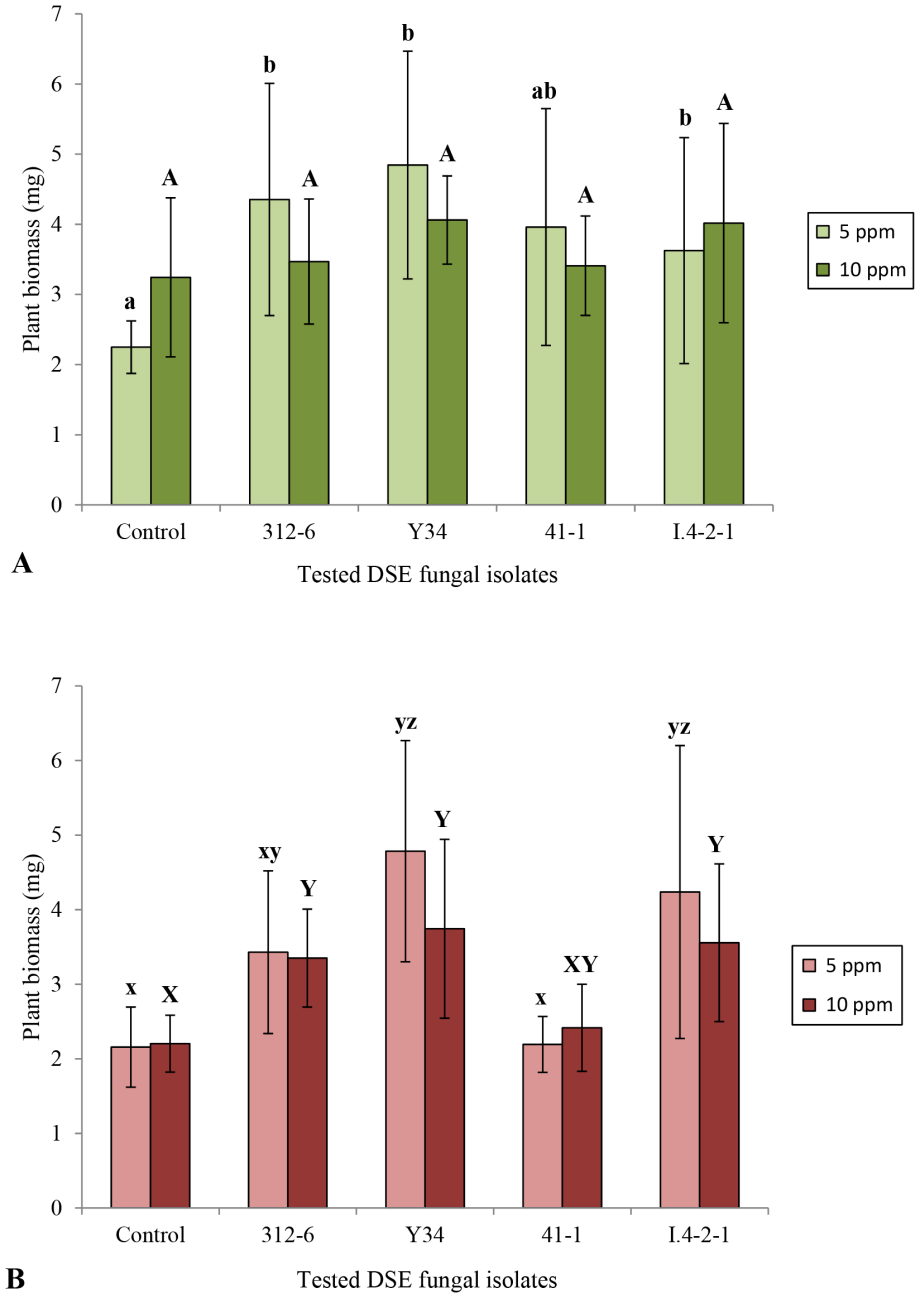
Effect of DSE fungal isolates on plant growth under cesium contamination conditions. (A) Dry weight of Chinese cabbage seedlings grown on oatmeal medium enriched with MgSO_4_.7H_2_O, KH_2_PO_4_, and Nature Aid, supplemented with cesium (concentration 5 ppm and 10 ppm) and inoculated with four selected isolates (Control: without fungal isolate). Data are the mean ± SD, n = 7. Within the same concentration, columns with the same letters are not significantly different at P<0.05. (B): Dry weight of tomato seedlings.

Chinese cabbage seedlings inoculation with DSE isolates showed improved plant biomass of 49%, 64% and 82% with isolates *P. ibarakiensis* isolate I.4-2-1, unidentified taxon isolate 312-6, and *V. simplex* isolate Y34, respectively under Cs [5ppm], whereas under Cs [10ppm], no increased biomass was observed (Fig. 1A).

On the other hand, tomato seedlings showed an increased biomass of 96% and 122% when treated respectively with *P. ibarakiensis* isolate I.4-2-1, and *V. simplex* isolate Y34 under Cs [5ppm], whereas under Cs [10ppm], tomato inoculated with isolate Y34 showed an increased biomass of 70% (Fig. 1B).

### Cs content per dried plants in DSE-treated Chinese cabbage tomato seedlings

**Figure 2:**
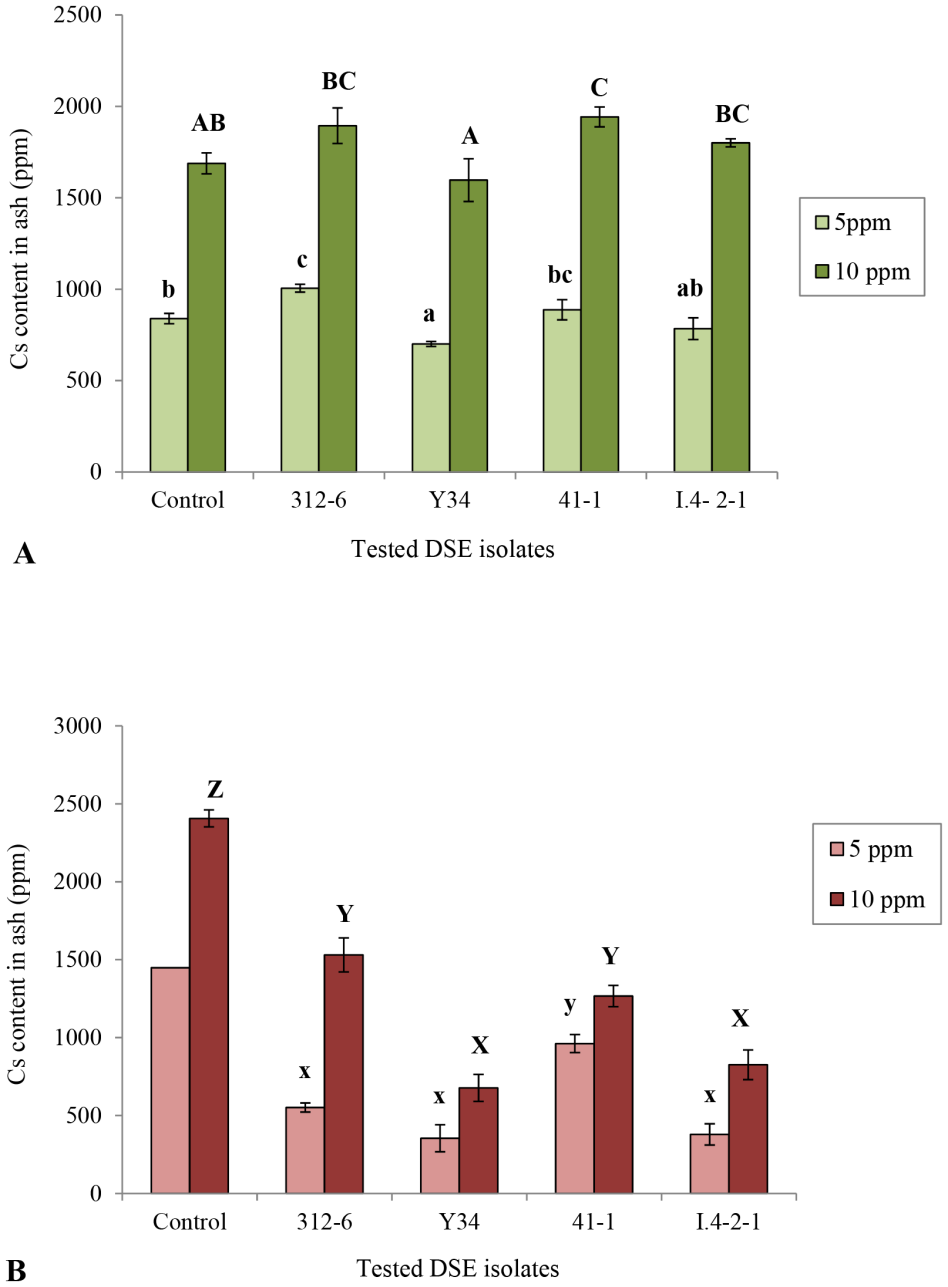
Effect of DSE fungal isolates on the cesium accumulation in host plants. (A) Cesium contents in ash (ppm) of Chinese cabbage seedlings grown on oatmeal medium enriched with MgSO_4_.7H_2_O, KH_2_PO_4_, and Nature Aid, supplemented with cesium (concentration 5 ppm and 10 ppm) and inoculated with four selected isolates (Control: without fungal isolate). (B) Estimated cesium content per dried plant (ppm) in tomato seedlings.

The content of Cs in dried Chinese cabbage seedlings increased with all DSE fungal isolates compared to control plants reaching 100% with isolate 41-1 in Cs [5ppm] and 129% with isolate I.4-2-1in Cs [10ppm] (Fig. 2A).

In tomato, this Cs content per dried plants has decreased significantly compared to control plants, reaching 73% and 79% with isolates *V. simplex* isolate Y34, and yet unidentified isolate 312-6, respectively, in Cs [5ppm]. Under Cs [10ppm], the content was reduced by 75% with isolate Y34 (Fig. 2B).

### Anatomic observation

**Figure 3:**
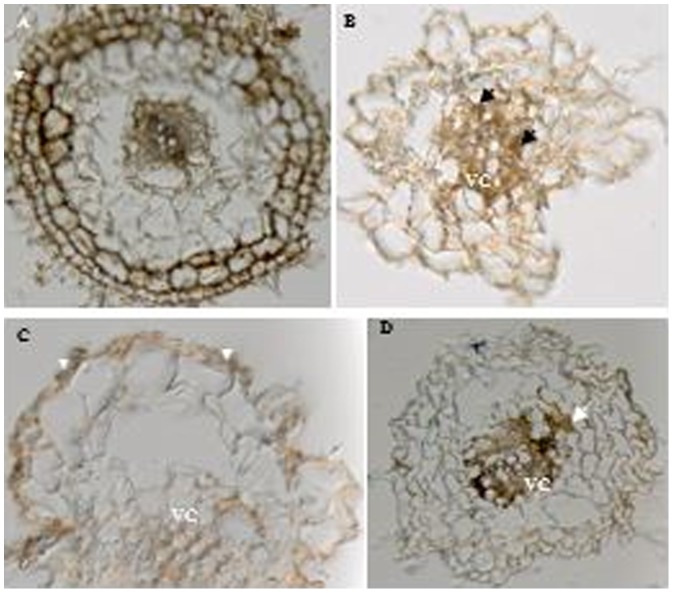
Interactions between dark septate endophytic fungus *Pseudosigmoidea ibarakiensis* isolate I.4-2-1 and roots of two host plants. (A, C) Cross sections of a tomato and a Chinese cabbage root segment stained with 0.005% cotton blue in 50% acetic acid three weeks after inoculation showing fungal hyphae on the root surface, within epidermal cells (EP) (head arrows). VC = vascular cylinder. (B,D) Cross sections of an un-inoculated tomato and a Chinese cabbage root segment serving as controls and showing disruption of vascular cylinder (arrows).

After 3 weeks of incubation, I.4-2-1 was successfully established in tomato and Chinese cabbage seedlings without any visible signs of either the host reactions or disruptions in the root cells colonized by the fungus (Fig. 3A&C). In the un-inoculated controls, most of the vascular cylinder cells were disrupted during the observation periods (Fig. 3B&D).

## Discussion

The cycling of the radionuclides is not well understood in natural ecosystems due to high diversity [19]. It is well known that inoculation of endophytic bacteria to plants can improve biomass production and uptake/accumulation of Cs in contaminated environments [20]–[22]. However, although soil fungi have potential in radionuclide bioaccumulation [23], [24], how DSEs interact with plants with regard to growth and the accumulation of radioactive Cs in contaminated environments is poorly understood. In plants, Cs, especially at high levels, can affect plant growth by causing various negative effects, i.e., chlorosis, necrosis, phytotoxicity, reduction of root growth, etc. [25], [26].

In the present study, we demonstrated that DSE fungi also have the ability to enhance Chinese cabbage and tomato plants growth under Cs-contaminated conditions. Previously, some arbuscular mycorrhiza such as *Glomus mosseae* or *Rhizophagus irregularis* (*Glomus intraradices*) have been reported to promote the biomass of grass and sunflower species [11], [13] in Cs-contaminated environments. Endophytic bacteria also have been reported enhancing hyperaccumulator plants [27], [28]. Beside the use of plants and associated microorganisms, other techniques such as the use of elevated CO_2_ were developed to trigger hyperaccumulation of Cs in sorghum [29].

Therefore, to our knowledge, this study is the first report involving DSEs in such environments. Three of the isolates used in the present study, i.e., *H. velutinum* isolate 41-1, *V. simplex* isolate Y34 and *P. ibarakiensis* isolate I.4-2-1, have already been reported to support plant growth under nutrient-deficient and other acidic pH conditions but not in contaminated conditions [30], [31], [32]. These results shed a light on a new field of use of these isolates. Interestingly, the biomass increase achieved with the present DSEs is greater than the 41.7% to 71.7% increase obtained with arbuscular mycorrhizal fungi [11]. Their ability to support plants growth combined to the high accumulation capacity they conferred to host plants suggest increased risk of contamination in the food chain, as the concentration of Cs in this food chain is of key importance for human health [33], [34]. However, they can be a viable means for solving the problem of small biomass production which is one of the major limiting factors for using tolerant plants in bioremediation [35], [36]. These DSEs can be selected as potential candidates to undergo further studies in real life conditions before they can be recommended (if conclusive) to farmers in the surrounding contaminated areas. Considering that Brassica plants are easily grown crops, their combination with DSEs can enhance phtyoremediation by means of Brassica plants including at least Chinese cabbage for extracting the Cs from contaminated soils. The DSE can even live in symbiosis with non-mycorrhizal plants such as Brassica plant at the present state [16]. We did not extend our study to other host plants and DSE species; however, other DESs might share the same abilities. Until now, DSE fungi in general have been mainly known to support plant growth under unfavorable conditions [14], [37].

Furthermore, our study demonstrated consistently the ability conferred to tomato plants by DSEs to decrease the accumulation of Cs under laboratory conditions. Previously, some studies reported improved uptake and/or accumulation of Cs by host plants using mycorrhiza, not DSE fungi [38], [39], [13]. For example, plant colonization by AM can increase Cs uptake [40]. Thus, to our knowledge, this study is the first report of DSE improving (decreasing) host Cs accumulation in contaminated environments.

These results demonstrate how DSE fungal symbionts are likely to function in radionuclide-polluted environments by regulating the bioavailability of Cs, confirming the hypothesis of the multifunctionality of DSE-plant symbioses [41]. The impact of these interactions goes beyond nutrient acquisition and the resultant positive host growth responses.

We are not able to explain the exact mechanism by which DSEs can promote plant growth or decrease the accumulation of Cs by their host plants, however, it might be related either to the sequestration of Cs in fungal hyphae or to the effects of ionizing radiation on the fungus. Exposure to ionizing radiation was recently proved to enhance the growth of melanized fungi [42]. The good performance of DSEs that also possess this melanin pigment in Cs-contaminated environments may also be related to this phenomenon. More studies are needed to support such hypotheses.

This finding is important as it shows another function of DSE fungi. Previously, these fungi were known to improve plant fitness through nutrient transfer and so on [16], [18]. This finding suggests that DSEs tend to display a multifunctional symbiosis with host plants, as is the case for mycorrhizal fungi and their host plants. These results can be used as a model in attempts to solve the crucial food chain contamination caused by these radionuclides. They can mainly be recommended to tomato growers in contaminated areas to reduce in a sustainable way the accumulation of Cs in the primary food chain by preceding tomato cultivation by Chinese cabbage cultivation as tomato represents a high demand for consumers. For future and more scientific perspectives, they can also serve as a starting point for more detailed investigations to clarify the mechanisms underlying beneficial plant-fungus interactions in DSE symbiosis.
